# Effect of Poly-Histidine Tag Position toward Inhibition Activity of Secretory Leukocyte Protease Inhibitor as Candidate for Material Wound Healing

**Published:** 2020

**Authors:** Elly Munadziroh, Evi Umayah Ulfa, Amaliah Labiqah, One Asmarani, Ni Nyoman Tri Puspaningsih

**Affiliations:** 1. Department of Dental Material and Technology, Faculty of Dentistry, Universitas Airlangga, Surabaya, Indonesia; 2. Department of Pharmaceutical Biology, Faculty of Pharmacy, Universitas Jember, Jember, Indonesia; 3. Department of Chemistry, Faculty of Science and Technology, Universitas Airlangga, Surabaya, Indonesia; 4. Department of Health Analysis, Stikes Kesetiakawanan Sosial Indonesia, Jakarta, Indonesia; 5. Proteomic Study Group, Institute of Tropical Disease, Universitas Airlangga, Surabaya, Indonesia

**Keywords:** *Escherichia coli*, Gene expression, Poly histidine, Secretory leukocyte protease inhibitor

## Abstract

**Background:**

The Secretory Leukocyte Protease Inhibitors (SLPI) has many biological functions including anti-bacterial, anti-fungal, anti-viral, anti-inflammatory, and immuno-modulatory. Previous studies have shown that gene-encoding human SLPI have successfully been expressed in *Escherichia coli (E. coli)* with a C-terminal poly-histidine tag (His-tag). The aim of this research was to investigate the inhibition activity of N-terminal His-tag position (NSLPI) and C-terminal His-tag position (CSLPI). We hypothesized that a His-tag close to an active site SLPI domain may interfere with the inhibition activity of SLPIs.

**Methods:**

A NSLPI and CSLPI were constructed with polymerase chain reaction (PCR) amplification. The PCR products were then ligated into pET-30a plasmid and transformed into *E. coli* TOP10. Recombinant plasmids were verified by using restriction analysis and nucleotide sequence analysis. pET-NSLPI and pET-CSLPI were then subcloned in *E. coli* BL21(DE3) for its expression. The SLPI protein was expressed using Isopropyl β-D-1-thiogalactopyranoside induction (IPTG). The inhibition effect of both SLPI against Porcine Pancreatic Elastase (PPE) enzyme was tested using the N-succinyil-alanyl-L-alanyl-L-prolyl-L-phenylalanyl-4-nitroanalide (NPN) substrate.

**Results:**

The SLPI gene was successfully cloned and expressed in *E. coli* BL21. Fusion proteins of NSLPI and CSLPI were generated with His-tag in the N-terminal and C-terminal position, respectively. The inhibition effect of NSLPI and CSLPI on PPE indicated that both SLPI were active. The inhibition activity of NSLPI was 66.7%, higher than CSLPI by 44.4%.

**Conclusion:**

The His-tag position on the C-terminal of SLPI reduced the inhibition activity of SLPI.

## Introduction

The Secretory Leukocyte Protease Inhibitor (SLPI) is a small protein (11.7 *kDa*) composed of two Whey Acidic Protein (WAP) domains. An SLPI is expressed mainly in mucosal secretion, including nasal, bronchial, tear, cervical, amnion, and seminal plasma 1. The pivotal role of an SLPI is to protect epithelial tissue during inflammation by inhibiting proteases, which are involved in degradation tissues (*e.g*., chymotrypsin, trypsin, neutrophil elastase, and cathepsin G) 2. However, recent studies have indicated numerous functions of SLPI aside from just protease inhibitor.

An SLPI possesses antimicrobial activity against gram-positive and gram-negative bacteria, mycobacteria, and fungi. SLPI also controls TGFβ expression, causing a reduced inflammatory response 3, can inhibit the transmission of HIV-1 4, and promotes wound healing 3,5. SLPI prevents the release of cytokines (such as interleukin -1β and interleukin-10) and the increase of matrix metalloproteinase by inhibiting the activation of nuclear factor kB 6–8. Moreover, SLPI has reduced reactive oxygen species production from H_2_O_2_-induced cardiac fibroblast 9. Based on anti-microbial, anti-oxidant, immunomodulatory, wound healing, and anti-inflammatory properties, SLPI can be recruited as a promising candidate for many types of therapy.

An SLPI’s structure consists of two domains. Each domain contains eight cysteine residues that form four disulfide bonds and stabilize the domain structure 10. This cysteine-rich domain, also referred as the WAP domain due to the motif signature describing the position of the conserved cysteine and the location of the disulfide bonds, was first identified in WAP-abundant in rodent milk 2. Based on the crystal structure of SLPI with various serine proteases, it can be concluded that inhibition activity against serine proteases is located in the C-terminal domain, while the N-terminal domain of SLPI is responsible for anti-microbial activities and stabilized the protease inhibitor complex 10–12.

In our previous study, the SLPI gene from amniotic membranes were cloned and expressed in *Escherichia coli (E. coli)* BL21 (DE3) by using pET101/DTOPO, which produced active recombinant human SLPI. This protein contains a native signal peptide and poly-histidine tag (His-tag) in the C-terminal of SLPI 13. His-tags are used to simplify the recombinant proteins purification using immobilized metal affinity chromatography 14. His-tags can be placed to the C-terminal or N-terminal of a recombinant protein. Even though it is possible to remove affinity tags using several methods, small tags are frequently left on the protein after the reaction, which lead to several drawbacks (*e.g*., it exhibits low efficiency, yet needs a large amount of enzymes) 15. Commonly, His-tag addition on recombinant proteins does not alter the protein properties and functions. However, in another cases 16–19, His-tag position cause alterations in protein structure and activity.

In this study, we have engineered SLPI with N-terminal His-tag (NSLPI) and C-terminal His-tag (CSLPI). We have also investigated the effect of His-tag positions on the activity of SLPI. We hypothesized that the position of the His-tag in such close adjacent proximity to the C-terminal has potential to reduce the activity of SLPI.

## Materials and Methods

### Materials

*E. coli* strain BL21 Star (DE3) harboring pET-TOPO-ESLPI derived from previous work was used for gene manipulation. The *E. coli* TOP10 (Invitrogen, Carlsbad, CA, USA) and BL21 (DE3) (Novagen, Darmstadt, Germany) strains were used as a cloning host and an expression host, respectively. pET30a (Novagen) plasmids were used for the construction of the expression system. The cloning process used restriction enzymes, such as *Nde*I, *Xho*I, *EcoR*I, and DNA ligase (Thermo Fisher Scientific, USA). The primers were synthesized by IDT. DNA was extracted and purified using a QIAamp Kit (Qiagen, Carlsbad, CA, USA). Luria-Bertani (LB) medium supplemented with kanamycin (50 μ*g ml*^−1^) and Isopropyl β-D-1-thiogalacto-pyranosides (IPTGs) were used for the growth of recombinant bacteria and protein expression. Porcine Pancreatic Elastase (PPE) and N-succinyil-alanyl-L-alanyl-L-prolyl-L-phenylalanyl-4-nitroanalide (NPN) (Sigma, St. Louis, MO, USA) were used as enzyme and substrate, respectively, to examine inhibitor activity.

### Construction of pET-NSLPI, and pET-CSLPI Recombinant Plasmids

A pET-ESLPI containing a full-length SLPI coding sequence was used as the template for polymerase chain reaction (PCR) amplification. A pET-NSLPI was generated by PCR amplification using two primers: 5′-GATTA<u>GAATTC</u>ATGAAGTCCAGCGGCCTCTTCCC-3′ and 5′-GCCTCGAGTCAACCGGTACGCGTA GAATC-3′. These primers consist of recognition sites of *EcoR*I and *Xho*I restriction enzymes (underlined). In pET-CSLPI construction, PCR amplification was completed with a sense *Nde*I-linker primer (5′GC<u>CATATG</u> ATGAAGTCCAGCGGCCTCT3′) and an antisense *Xho*I-linker primer (5′CGAC<u>CTCGAG</u>TCAATGGTG ATGGTGATGATGAC-3′). The PCR products were analyzed in 1% agarose gel electrophoresis and purified. The sizes of PCR products were 477 *bp* and 495 *bp* for NSLPI and CSLPI, respectively.

All recombinant plasmids were constructed from the plasmid of pET30a. To construct pET-NSLPI vectors, the plasmid pET30a was digested with *EcoR*I and *Xho*I, then ligated to the NSLPI gene. By contrast, the design of the pET-CSLPI vectors used the plasmid pET30a digested with *Nde*I and *Xho*I before being ligated with CSLPI gene. The ligation process used the compatible sticky-end DNA molecules for both plasmid pET30a (5400 *bp*) and the genes (NSLPI and CSLPI). Ligated mixtures were transformed into *E. coli* TOP10 and the recombinant plasmid were selected on LB media supplemented with 50 *μg/ml* kanamycin plates. Nucleotide sequences of the plasmids were confirmed by sequencing.

### Cloning and Expression of SLPI Genes

For protein expression, each constructed plasmid was transformed into *E. coli* BL21. The plasmid, without SLPI genes as a control, was also transformed into these bacterial strains. A single colony harboring each specific plasmid was taken from the transformation plate and inoculated into LB liquid culture supplemented with 50 *μg/ml* kanamycin. The bacteria were grown at 37*°C* and inducted by 0.6 *mM* IPTG. The cell was subsequently harvested three hours after IPTG induction. The cell was disrupted by sonication, and then the protein expression was analyzed by sodium dodecyl sulfate polyacrylamide gel elelctrophoresis (SDS-PAGE). A western blot analysis using a monoclonal rabbit antibody against SLPI (Santa Cruz Biotechnology) was performed to confirm the identity of the SLPI. The SLPI was visualized with conjugate anti-rabbit alkaline phosphatase (Promega, Madison, WI, USA).

### SLPI inhibition assay

SLPI was tested for inhibition activity on PPE. Briefly, 600 μl of 0.2 *M* Tris-HCl pH=8.8, 100 *μl* of 0.1 *mg/ml* PPE, and 25 *μl* of SLPI were added to a microcentrifuge tube. Then, 750 *μl* of NPN substrate was added and mixed gently. The absorbance of p Nitro-Aniline (pNA) product was measured at 410 *nm* every 30 *s* for four *min*. The activity of PPE was determined by replacing SLPI with buffer. The inhibition degree of the inhibitor was calculated using the following equation:

$$i=\frac{\text{v}0-\text{v}1}{\text{v}0}$$

where *i* was the degree of inhibition; V_0_ was the reaction rate without inhibitor; and V_1_ was the reaction rate in the presence of inhibitor. The total protein of *E. coli* pET 30a was used as control.

## Results

### Construction of pET-NSLPI and pET-CSLPI Recombinant Plasmids

**Figure 1.**
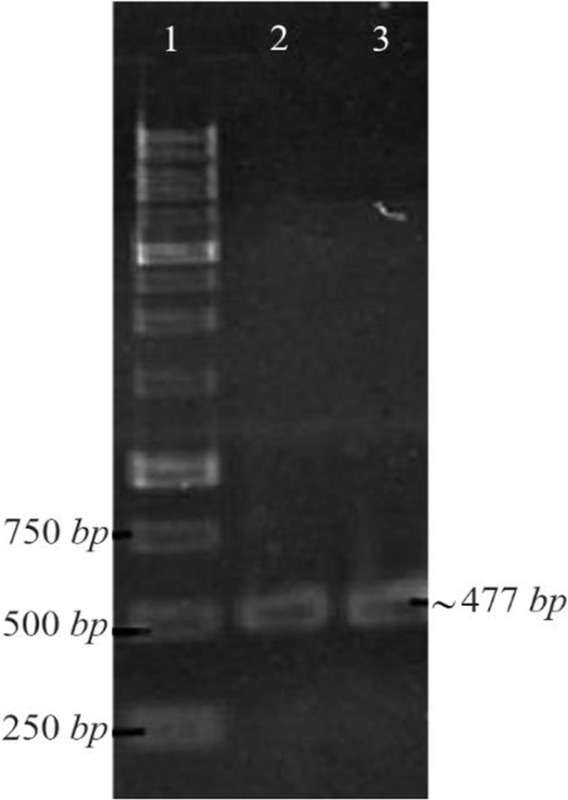
PCR amplification of pET-ESLPI for generating NSLPI and CSLPI: (1) 1 *Kb* DNA ladder, (2) PCR product of NSLPI, (3) PCR product of CSLPI.

NSLPI and CSLPI were successfully amplified using Taq DNA polymerase (Thermo Fisher Scientific, USA) from pET-ESLPI recombinant plasmid 13. An electrophoregram of PCR products is shown in figure 1 as a single band approximately 477 and 495 *bp*, respectively, similar to the theoretical size.

**Figure 2.**
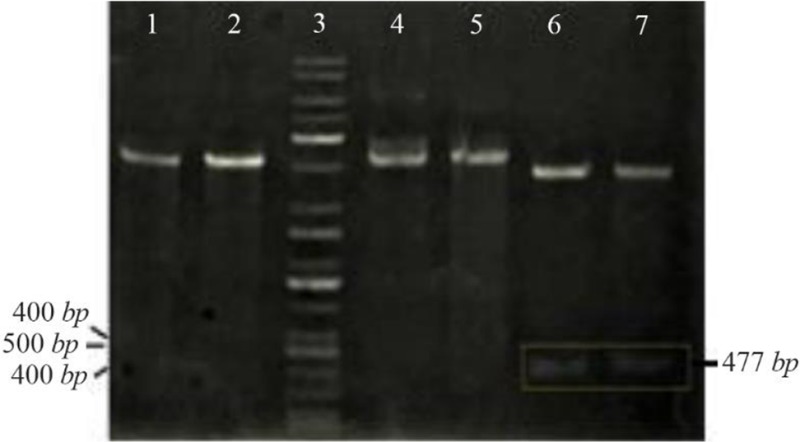
Restriction analysis of recombinant plasmid: (1) pET-NSLPI digested by *Xho*I, (2) pET-CSLPI digested by *Xho*I, (3) 1 *kb* DNA ladder, (4) undigested pET-NSLPI, (5) undigested pET-CSLPI, (6) pET-NSLPI digested by *Xho*I and *Eco*RI, and (7) pET CSLPI digested by *Nde*I and *Xho*I.

The PCR products (NSLPI and CSLPI) were digested and ligated into a pET30a vector. Plasmid from several colonies, which were grown on LB medium containing kanamycin, was isolated and analyzed using single and double digestion to confirm the presence of the SLPI gene. A single band about 5.9 *kilobases* (*kb*) in size was obtained after a single digestion of recombinant plasmid. A double digestion of pET-NSLPI and pET-CSLPI recombinant plasmids, displayed in figure 2, showed two DNA bands consisting of vector 5.4 *kb* (pET30a) and the insertion of 0.5 *kb* (SLPI). This data indicated that the pET30a successfully harbored the SLPI gene with His-tag on the N-terminal and C-terminal positions. The sequencing results on the recombinant plasmid showed that pET-NSLPI contained the SLPI gene present in-frame with a His-tag on the N-terminal, while pET-CSLPI containing the SLPI gene was present in-frame with a His-tag on the C-terminal. A sequence alignment analysis of NSLPI and CSLPI was compared to a SLPI gene retrieved from Genebank and revealed 100% homology. A basic local alignment search tool analysis on the amino acid sequence showed 100% similarity with the amino acid of SLPI from a human.

### Expression of NSLPI and CSLPI

**Figure 3.**
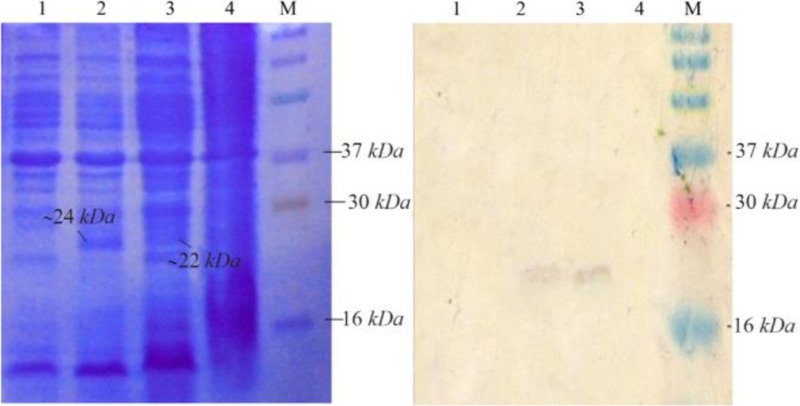
Analysis of SLPI expression. Coomassie blue stained 15% SDS-PAGE (A), western blot analysis. M. Molecular weight marker, (1) total protein of *E. coli* pET-NSLPI without IPTG induction, (2) total protein of *E. coli* pET-NSLPI under 0.6 *mM* IPTG induction, (3) total protein of *E. coli* pET-CSLPI under 0.6mM IPTG induction, and (4) total protein of *E. coli* pET-CSLPI without IPTG induction.

NSLPI and CSLPI were produced as a fusion protein with His-tag. The additional amino acid residues at the N-terminal of SLPI were MHHHHHHSSGLVPRG SGMKETAAAKFERQHMDSPDLGTDDDDKAMA DIGSEF. The molecular weight of NSLPI was about 24 *kDa*, consisting of 18 *kDa* of SLPI and 6 *kDa* of additional protein. That being said, the CSLPI had 32 additional amino acid residues (KGELNSKLEGKPIPNPLLGLDSTRTGHHHHHH) at the C-terminal of SLPI. The molecular weight of CSLPI was ±22 *kDa*. An SDS-PAGE analysis of the total intracellular protein showed the presence of a protein band at ±24 *kDa* (NSLPI) and ±22 *kDa* (CSLPI). This size appeared to correspond to the theoretical size of NSLPI and CSLPI with additional His-tag (Figure 3).

### Biological activity of NSLPI and CSLPI

**Figure 4.**
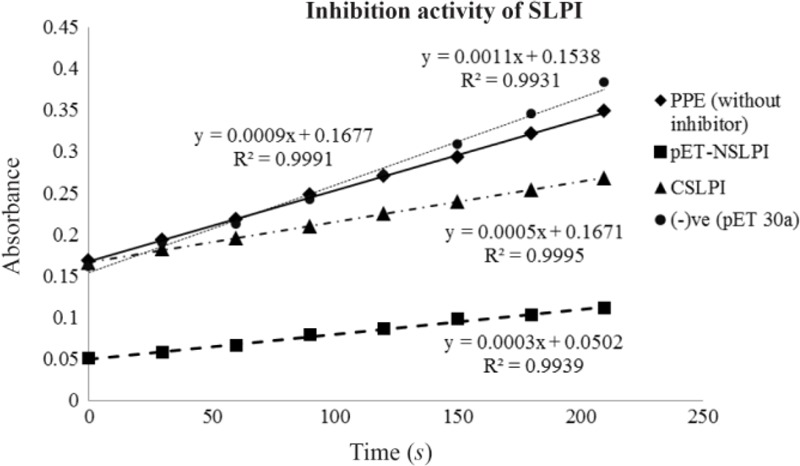
Inhibition activity of NSLPI and CSLPI against PPE.

The inhibition activity of NSLPI and CSLPI against PPE were examined by using NPN substrate. Our previous study demonstrated that rSLPI blocks NPN cleavage by PPE 13. The interaction between rSLPI and PPE causes the amount of p-NA released during NPN substrate hydrolysis to decrease. As seen in figure 4, NSLPI and CSLPI inhibited the PPE.

The percent inhibitions of NSLPI and CSLPI were 66.7 and 44.4%, respectively, indicating the ability of SLPI to inhibit the action of PPE. The percent inhibition of CSLPI was 44.4%, similar to previous study reported by Purnamasary (45%) 19. Based on the value of the percentage inhibition, NSLPI showed higher inhibition activity against PPE than CSLPI.

## Discussion

In this study, we demonstrated that His-tag position affects the inhibition activity of SLPI against PPE. The His-tags at C-terminal and N-terminal provide different inhibitory activities. Inhibition activity of CSLPI against PPE is lower than NSLPI. The location where SLPI primary reacts with serine protease is amino acid residue, G69-Q70-C71-L72-M73-L74, on the C-terminal domain of SLPI 10. The His-tags placed on the C-terminal of SLPI is speculated to interfere with protein properties due to steric or electrostatic interaction of His-tag. The electrostatic interaction may result from positively charged histidines.

The effect of His-tag position for protein activity was also investigated by Yeon 20. A His-tag located near the active site of 3-hydroxybutyrate dehydrogenase (3HBDH) can separate hydrogen bonds, disturb the conformation of 3HBDH, and decrease the catalytic enzyme. On the other study 16, it is also reported that His-tag in the position at C-terminal of tropinone reductase interfered the active site of the enzyme. The results confirmed that the functional characterization of recombinant proteins using affinity tags is necessary before further applications can be pursued. Further in-vestigation using protein structure modeling is necessary to clarify possible interaction of His-tag with active site of recombinant protein.

## Conclusion

The gene-encoding SLPI has been successfully constructed and expressed its recombinant with a His-tag on either the N-terminal or C-terminal in an *E. coli* system by using 0.6 *mM* IPTG. The His-tag attached at the C-terminal of SLPI reduced the inhibition activity of SLPI.
